# NZ-RugbyHealth Study: Self-reported Injury Experience and Current Health of Former Rugby Union and Non-contact Sport Players

**DOI:** 10.1007/s40279-021-01630-7

**Published:** 2022-01-28

**Authors:** Patria A. Hume, Kenneth L. Quarrie, Gwyn N. Lewis, Alice Theadom

**Affiliations:** 1 Sport Performance Research Institute New Zealand, Faculty of Health and Environmental Sciences, Auckland University of Technology, Private Bag 92006, Auckland, 1142 New Zealand; 2 Traumatic Brain Injury Network (TBIN), Auckland University of Technology, Auckland, New Zealand; 3 Health and Rehabilitation Research Institute (HRRI), Auckland University of Technology, Auckland, New Zealand; 4 New Zealand Rugby, Wellington, New Zealand

## Abstract

**Background:**

There is limited research on associations between playing rugby union and player health post-retirement.

**Objective:**

This study investigated differences in self-reported sport injury history and current self-reported health characteristics between former New Zealand rugby and non-contact sport players with a view to identifying issues to be further investigated with stronger epidemiological research designs.

**Methods:**

Using a cross-sectional design, the *NZ-RugbyHealth* study surveyed 470 former rugby and non-contact sport players (43.8 ± 8.1 years; 127 elite rugby, 271 community rugby, 72 non-contact sport) recruited from October 2012 to April 2014. Demographic information, engagement in sport, sport injuries, medical conditions, mood, alcohol and substance use and ratings of current health status were obtained from a self-report 58-item general health e-questionnaire. We highlighted standardised differences in means of > 0.6 and differences in relative percentages of > 1.43 for variables between groups as representing at least moderate effect sizes, and of being worthy of follow-up studies.

**Results:**

Higher percentages of the elite rugby player group had sustained injuries of a given body-site type (e.g. neck sprain/strain, thigh bruising, hamstring strain) combination than the non-contact sports players. Higher percentages of the rugby groups reported having sustained concussion (94% for elite, 82% for community, 26% for non-contact), injuries requiring hospitalisation (73%, 46%, 25%), injuries that stopped participation in sport permanently (28%, 28%, 11%) and sport-related surgery (72%, 46%, 32%) during their playing career. Both rugby groups had a higher prevalence of osteoarthritis (37%, 18%, 6%) than non-contact athletes and community rugby players had higher levels of hazardous alcohol consumption (38%, 40%, 25%) in retirement than non-contact athletes. There was little difference between rugby players and non-contact sports athletes in self-reported mood, substance use and current physical or psychological health ratings.

**Conclusions:**

Former rugby player groups were at higher risk than the non-contact player group for most injuries during their playing careers, and in retirement had greater prevalence of osteoarthritis and hazardous alcohol consumption. The relative youth of the groups (43.8 years on average) means that health issues that typically do not emerge until later life may not have yet manifested.

**Supplementary Information:**

The online version contains supplementary material available at 10.1007/s40279-021-01630-7.

## Key Points

**Table:** 

Community and elite former rugby union players reported a substantially higher number of concussions, injuries requiring hospital treatment and injuries that stopped participation in sport permanently, than non-contact sport players.
A greater percentage of former rugby players reported that they had osteoarthritis and consumed alcohol at more hazardous levels compared with former non-contact sport players.
There was little difference between rugby players and non-contact sports athletes in self-reported mood, substance use, or current physical and psychological health ratings.

## Introduction

High impact loads in sport can result in injuries [1], along with potential reductions in health-related quality of life, post-retirement from sport [2–5]. The New Zealand RugbyHealth (*NZ-RugbyHealth)* project, containing four studies [6], was developed in 2012 at the request of World Rugby and conducted by New Zealand Rugby in collaboration with Auckland University of Technology researchers. The purpose of the project was to describe the injuries sustained by players during their playing careers, along with self-evaluations of their health after retiring from their sport.

In 1993, a prospective cohort study of the behaviours, attitudes and injury experience of club rugby players was conducted in Dunedin, New Zealand. The “Rugby Injury and Performance Project” (RIPP) [7–10] surveyed 258 male players (20.6 ± 3.7 years old) about their previous experiences with rugby and injury, their attitudes regarding injury prevention, their mood and typical ways of expressing anger and their alcohol and drug use at the beginning of the 1993 rugby season. Significant findings from RIPP were that the tackle was the aspect of rugby associated with the greatest proportion of injuries, that 13% of match injuries were the result of foul play, and that significant numbers of players engaged in binge drinking of alcohol weekly or more often (mean Alcohol Use Disorders Identification Test [AUDIT] score of 11.2 ± 5.1). The patterns of drinking exhibited by the cohort gave cause for concern at the time regarding the health risks associated with such behaviour [11]. The methods from the 1993 RIPP study informed the methods of the present study. Where possible, we used the same questions in both studies to enable discussion of changes over the 20 years between the two studies, particularly given the findings from RIPP formed a major part of subsequent injury prevention efforts in New Zealand rugby. These changes included the development of RugbySmart, adapted from SportSmart [12], and research into aspects of tackles that modify injury risks to players [13].

This paper, therefore, describes the self-reported playing injury history and post-retirement physical, mental and social health characteristics of former elite rugby, community rugby and a comparison group of non-contact sport players. The findings were intended to facilitate the development of hypotheses between exposure to rugby and health outcomes to enable investigation with stronger epidemiological research designs.

## Methods

### Study Design and Setting

*NZ-RugbyHealth* was a cross-sectional study of former elite and community rugby players and a non-contact-sport comparison group. To protect the identity of athletes, the study was completed anonymously online. Participants gave informed consent after reading a participant information sheet about the project. Ethical approval for the study was obtained from an institutional ethics committee (AUTEC #12/252). Our participant recruitment from October 2012 to April 2014 using media reports, word-of-mouth, flyers and social media resulted in the sample size being met for the community rugby group, but below the expectation of 200 per group for the elite rugby and non-contact sport group.

### Participants

*NZ-RugbyHealth* [6] participants were 470 former (retired from competitive play) New Zealand male athletes drawn from three groups: 127 elite rugby (international or national level), 271 community rugby (club or regional level) and 72 non-contact sport (cricket or hockey players at any level). Elite players are what would now be termed professional players; however, when many of the older participants in the study played at the international or national level of rugby, it was still amateur rugby given players were not paid to play. A non-contact sport participation group was included as we were particularly interested at the start of the study in comparing the injury history and current physical and mental health of those exposed to sport involving physical contact (rugby players) with those exposed to sport that does not involve collision. The groups were matched to be of similar age and to have similar exposure time to sport, with the non-contact group covering a range of levels from community to elite. Thus, the inclusion of the non-contact control group enabled a comparison of current and former health between retired athletes who were and were not exposed to physical contact in their sport.

### Procedures

Information on engagement in sport, sport-related injury, demographic information, current physical health (including engagement in activities of daily living), current mental health and current health behaviours (including levels of smoking, alcohol and drug use) was elicited from a self-report rugby-sport general health 58-item e-questionnaire developed for the study. Where possible, items in the questionnaire were based on other, validated questionnaires, as described in following sections. The research team had the opportunity to use some of the same RIPP questionnaires [9] to determine health and lifestyle patterns, playing experience and injury experience. The *NZ-RugbyHealth* questionnaire took approximately 40 min to complete.

### Study Sample

Demographic information included self-reported years of sport played, games played, ethnicity, highest education qualification and current age, height, weight (allowing body mass index calculation), annual income, occupation and marital status. Ethnic groups were categorised as Māori (indigenous people to New Zealand), Pākehā (New Zealand European), Pasifika (Samoan, Fijian, Tongan and other Pacific Island descent) and other.

### Playing Career Injuries and Illnesses

Sport-related injury and illness history questions included the number of injuries sustained while playing sport, number of injuries requiring hospitalisation, number of surgeries from a sports injury, whether an injury had stopped participation in sport permanently and whether a medical condition (e.g. diabetes) had developed while playing sport.

History of concussion was determined by asking participants several questions about their experiences of concussion. For example, they were asked how many times they had sustained a concussion whilst playing or training for rugby/hockey/cricket, had been evaluated by a doctor or other health professional for concussion, had lost consciousness (been ‘knocked out’) or sustained other symptoms of concussion. Concussion was defined as being a blow to the head followed by a variety of symptoms (loss of consciousness, headache, dizziness, loss of balance, blurred vision, ‘seeing stars’, feeling in a fog or slowed down, memory problems, poor concentration, nausea, or throwing up).

### Current Health

Participants were asked to rate their current overall health, physical health, mental/psychological health, diet/nutritional habits and how physically fit they are compared with other people their age on a Likert scale (available responses from 1 ‘very poor’ to 5 ‘excellent’). Participants were also asked to rate how intense their typical exercise was from 0 ‘very low intensity’ to 10 ‘very high intensity’.

Participants were asked whether they had been (either previously or currently) diagnosed by a health professional or taken medication for each of the following conditions: diabetes, chronic fatigue, cardiovascular disease, high blood pressure, high cholesterol, osteoarthritis, depression or bipolar disorder, anxiety, regular headaches/migraine, Alzheimer’s disease, Parkinson’s disease, dementia, or other. Having received either a diagnosis or having been prescribed medication for a given condition, either currently or previously, was coded as a positive response for that condition. There were no positive responses for Parkinson’s disease, and only one across the three groups for either dementia or Alzheimer’s, so no analyses were conducted for these conditions and they are not reported in the results.

Following the approach used in the RIPP [14], 16 items were taken from the Affectometer 2 0 questionnaire to assess affect (mood). The items consisted of eight phrases and eight single-word adjectives; four of each being positive phrases or words and four of each being negative. Respondents were asked to rate the degree to which the phrases or words applied to them over the past 4 weeks, on a scale from 1 ‘not at all’ to 5 ‘all the time’. The total possible score ranged from − 40 to + 40, with more positive scores indicating more positive affect. The sum of the scores on the negative items was subtracted from the sum of the scores on the positive items to yield a single score for affect.

Participant reactions and behaviour when angry were assessed using the Spielberger Anger Expression Scale [16] that consisted of 16 questions; eight measured the extent to which people internalised their feelings of anger (e.g. “I boil inside but don't show it”) and eight measured the extent they externalised their feelings of anger (e.g. “I do things like slam doors”). Each question was scored from 1 to 4 on a labelled four-point Likert scale, with the following answer options provided: ‘Almost never’, ‘Sometimes’, ‘Often’, ‘Almost always’. Possible scores ranged from 8 to 32 for each of the two subscales, ‘Anger In’ and ‘Anger Out’.

Alcohol use patterns were assessed using the Alcohol Use Disorders Identification Test (AUDIT) [17] that comprised three sub scales: consumption (items 1–3); Dependence (items 4–6); and Problems (items 7–10) [18]. Hazardous drinking was defined as a score > 8 on the AUDIT.

Participants were asked how often (never, previously, occasional, regularly) they had smoked tobacco or had used any non-prescription drugs (e.g. any use of cannabis in their lifetime).

### Statistical Analyses

Data were analysed using SAS/STAT version 9.4 and customised Microsoft Excel spreadsheets. Data are reported as mean ± standard deviation or a percentage (%) of participants, as indicated. Effect size statistics were calculated for comparisons across player groups (elite rugby, community rugby, non-contact sport). Comparative data from the literature was used for variable comparisons where possible. We have highlighted standardised differences in means that are > 0.6 (moderate effects or greater) in which the 95% confidence limits excluded zero, and relative percentages that are > 1.43 or < 0.70 where the relative percentage excluded 1.00 [19] between groups as representing at least moderate effect sizes. We believe these differences to be worthy of emphasising and following up with more robust research designs.

## Results

Table 1 displays the demographic and sports history characteristics of the study respondents. The average age of the 470 former players was 43.8 ± 8.1 years, with ages ranging from 29 to 75 years. There were differences between the rugby and non-contact groups for ethnicity and average body mass.

**Table 1:** Demographics for the former-player groups (elite rugby, community rugby, non-contact sport)

	Elite rugby		Community rugby		Non-contact sport		Elite rugby versus community rugby	Elite rugby versus non-contact sport	Community rugby versus non-contact sport
Numeric variables	*n*	Mean (SD)	*n*	Mean (SD)	*n*	Mean (SD)	Cohen's *d* (95% confidence limits)		
Age (years)	127	43 (8.5)	271	45 (8.0)	72	42 (7.5)	− 0.3 (− 0.5 to − 0.1)	0.0 (− 0.3 to 0.3)	0.3 (0.1 to 0.5)
Stature (cm)	126	184 (8.3)	267	180 (8.1)	71	181 (7.0)	0.5 (0.3 to 0.7)	0.4 (0.2 to 0.7)	− 0.1 (− 0.3 to 0.1)
Body mass (kg)	126	103 (15.4)	268	97 (18.1)	71	87 (10.6)	0.3 (0.1 to 0.5)	**1.2 (0.9 to 1.5)**	**0.6 (0.4 to 0.8)**
BMI (kg/m^2^)	126	30 (3.5)	267	30 (5.2)	71	26 (2.6)	0.1 (− 0.1 to 0.3)	**1.1 (0.8 to 1.5)**	**0.7 (0.5 to 0.9)**
Years of sport	123	23 (8.0)	264	23 (8.3)	70	25 (7.5)	0.0 (− 0.2 to 0.2)	− 0.2 (− 0.5 to 0.1)	− 0.2 (− 0.5 to − 0.0)

Percentage variables	*n*	%	*n*	%	*n*	%	Relative percentage (95% confidence limits)		
Played 150 matches or more	126	95	269	77	67	88	1.2 (1.2 to 1.3)	1.1 (1.0 to 1.2)	0.9 (0.8 to 1.0)
Ethnicity									
NZ European (Pākehā)	127	77	271	83	72	86	0.9 (0.8 to 1.0)	0.9 (0.8 to 1.0)	1.0 (0.9 to 1.1)
NZ Māori		20		16		2.8	1.2 (0.8 to 1.9)	**7.1 (1.7 to 29.1)**	**5.9 (1.5 to 23.5)**
Pasifika		13		3.0		0	**4.2 (1.9 to 9.7)**		
Other		6.3		6.3		11	1.0 (0.4 to 2.3)	0.6 (0.2 to 1.5)	0.6 (0.3 to 1.3)
Education									
Secondary school	127	23	271	28	72	21	0.8 (0.6 to 1.7)	1.1 (0.6 to 1.9)	1.4 (0.8 to 2.2)
Post-school diploma		22		28		12.5	0.8 (0.5 to 1.1)	**1.8 (0.9 to 3.5)**	**2.3 (1.2 to 4.3)**
Bachelor's degree		29		16		35	**1.8 (1.2 to 2.6)**	0.8 (0.6 to 1.3)	**0.5 (0.3 to 0.7)**
Post-graduate		21		18		31	1.1 (0.7 to 1.7)	0.7 (0.4 to 1.1)	**0.6 (0.4 to 0.9)**
Income p/a									
< $50,000	114	19	235	15	71	13	1.3 (0.8 to 2.1)	**1.5 (0.7 to 3.1)**	1.2 (0.6 to 2.3)
$50,000–$100,000		41		51		46	0.8 (0.6 to 1.0)	0.9 (0.6 to 1.2)	1.1 (0.8 to 1.5)
> $100,000		40		34		41	1.2 (0.9 to 1.6)	1.0 (0.7 to 1.4)	0.8 (0.6 to 1.2)
Marital status									
Married	122	92	246	90	71	87	1.0 (1.0 to 1.1)	1.1 (1.0 to 1.2)	1.0 (0.9 to 1.1)
Single		2.5		4.5		11	0.5 (0.2 to 1.9)	0.2 (0.1 to 0.8)	0.4 (0.2 to 1.0)
Divorced or widowed		5.7		5.7		1.4	1.0 (0.4 to 2.4)	**4.1 (0.5 to 32.4)**	**4.0 (0.5 to 30.2)**

Data are reported as mean ± standard deviation or a percentage (%) of participants as indicated. We have bolded standardised differences in means that are > 0.6 (moderate effects or greater) and relative percentages that are > 1.43 (at least a moderate difference between groups). The *n* is shown for the total group numbers who answered the questions for ethnicity, education, income per annum (p/a) and marital status

*BMI* body mass index, *NZ* New Zealand, *SD* standard deviation

Percentages of the former player groups having had at least one injury of a specific type from a list of 36 types, any sporting injury requiring hospitalisation or surgery, or an injury that stopped participation in sport permanently are presented in Table 2. The percentage of the elite player group reporting injuries of a given site–body region combination was uniformly higher than for the non-contact sports group (the difference was substantially greater, excluding a relative percentage of 1.0, for 44% of them) and, except for patellar dislocations, the community rugby player group (substantially greater for 50% of the site–body region combinations). A higher percentage of community rugby players reported injuries for most (73%) of the site–type combinations than did the non-contact group, with 19% of the differences being substantial.

**Table 2:** Percentages of the former player groups having had at least one injury of a specific type from a list of 36 types, any sporting injury requiring hospitalisation or surgery, or injury that stopped participation in sport permanently

	Elite rugby (*n* = 127) %	Community rugby (*n* = 271) %	Non-contact (*n* = 72) %	Relative percentages (95% CLs)		
				Elite versus community	Elite versus non-contact	Community rugby versus non-contact
Concussion	94	82	26	1.2 (1.1–1.2)	**3.6 (2.4**–**5.3)**	**3.1 (2.1**–**4.6)**
Facial/cheekbone/orbital/skull/nose fracture	57	41	14	**1.4 (1.1**–**1.7)**	**4.1 (2.3**–**7.4)**	**3.0 (1.7**–**5.4)**
Eye injury	26.8	25.1	16.7	1.1 (0.8–1.5)	**1.6 (0.9**–**2.9)**	**1.5 (0.9**–**2.6)**
Neck fracture/spinal cord injury	11.8	4.4	1.4	**2.7 (1.3**–**5.5)**	**8.5 (1.1**–**63.1)**	3.2 (0.4–24.1)
Neck sprain/strain	68.5	50.9	36.1	**1.4 (1.1**–**1.6)**	**1.9 (1.4**–**2.6)**	1.4 (1.0–2.0)
Clavicle fracture	19.7	13.3	8.3	1.5 (0.9–2.4)	2.4 (1.0–5.5)	1.6 (0.7–3.6)
Shoulder dislocation	25	17	11.1	**1.5 (1.0**–**2.2)**	**2.3 (1.1**–**4.7)**	1.5 (0.8–3.1)
Shoulder bruising	48	37.6	16.7	1.3 (1.0–1.6)	**2.9 (1.7**–**5.0)**	**2.3 (1.3**–**3.9)**
Shoulder strain	39.4	37.3	33.3	1.1 (0.8–1.4)	1.2 (0.8–1.7)	1.1 (0.8–1.6)
Shoulder sprain	37	21.4	6.9	**1.7 (1.3**–**2.4)**	**5.3 (2.2**–**12.8)**	**3.1 (1.3**–**7.4)**
Biceps/triceps tear	11	6.3	2.8	1.8 (0.9–3.5)	4.0 (0.9–17.0)	2.3 (0.5–9.6)
Elbow dislocation or separation	11.8	2.6	0	**4.6 (1.9**–**10.9)**		
Arm/wrist/hand fracture	47	31	39	**1.5 (1.2**–**2.0)**	1.2 (0.9–1.7)	0.8 (0.6–1.1)
Thumb or finger dislocation	46.5	32.5	30.6	**1.4 (1.1**–**1.8)**	1.5 (1.0–2.3)	1.1 (0.7–1.6)
Thumb or finger sprain	67.7	58.7	50	1.2 (1.0–1.4)	1.4 (1.0–1.8)	1.2 (0.9–1.5)
Disc rupture/herniation	19.7	7.4	12.5	**2.7 (1.5**–**4.6)**	1.6 (0.8–3.2)	0.6 (0.3–1.2)
Rib fracture	34	31	9.7	1.1 (0.8–1.5)	**3.5 (1.7**–**7.3)**	**3.2 (1.5**–**6.5)**
Rib bruising	53	40	25	1.3 (1.1–1.7)	**2.1 (1.4**–**3.3)**	1.6 (1.0–2.4)
Upper or lower back injury	61.4	46.9	55.6	1.3 (1.1–1.6)	1.1 (0.9–1.4)	0.8 (0.7–1.1)
Hip/pelvis dislocation or fracture	4.7	1.8	0	2.6 (0.8–8.2)		
Thigh/leg fracture	7.1	4.8	4.2	1.5 (0.7–3.4)	1.7 (0.5–6.1)	1.2 (0.3–3.9)
Thigh bruising	69.3	48.3	36.1	1.4 (1.2–1.7)	**1.9 (1.4**–**2.7)**	1.3 (1.0–1.9)
Thigh strain	31.5	18.5	29.2	**1.7 (1.2**–**2.4)**	1.1 (0.7–1.7)	0.6 (0.4–1.0)
Hamstring tear	40.3	23.2	29.2	**1.7 (1.3**–**2.3)**	1.4 (0.9–2.1)	0.8 (0.5–1.2)
Hamstring strain	60.6	42.4	56.9	**1.4 (1.2**–**1.7)**	1.1 (0.8–1.4)	0.8 (0.6–1.0)
Knee ligament rupture or tear	33.1	26.9	16.7	1.2 (0.9–1.7)	**2.0 (1.1**–**3.5)**	1.6 (0.9–2.8)
Knee ligament sprain	41.7	33.9	20.8	1.2 (0.9–1.6)	**2.0 (1.2**–**3.3)**	1.6 (1.0–2.6)
Knee patellar dislocation	4.7	5.2	2.8	0.9 (0.4–2.3)	1.7 (0.4–8.2)	1.9 (0.4–8.0)
Knee meniscus tear	33.9	21	13.9	**1.6 (1.2**–**2.3)**	**2.4 (1.3**–**4.6)**	1.5 (0.8–2.8)
Calf tear	33.9	14.4	19.4	**2.4 (1.6**–**3.4)**	1.7 (1.0–3.0)	0.7 (0.4–1.3)
Calf strain	59.1	33.9	43.1	**1.7 (1.4**–**2.2)**	1.4 (1.0–1.9)	0.8 (0.6–1.1)
Achilles tendon injury	22.8	11.8	13.9	**1.9 (1.2**–**3.1)**	1.6 (0.9–3.2)	0.9 (0.4–1.6)
Achilles tendon tear or rupture	7.9	4.4	1.4	1.8 (0.8–4.0)	5.7 (0.7–43.4)	3.2 (0.4–24.1)
Ankle ligament tear or rupture	29.9	15.1	12.5	**2.0 (1.3**–**2.9)**	**2.4 (1.2**–**4.7)**	1.2 (0.6–2.4)
Ankle ligament sprain	76.4	52.4	43.1	**1.5 (1.3**–**1.7)**	**1.8 (1.3**–**2.4)**	1.2 (0.9–1.6)
Ankle/foot fracture	18.1	10.7	13.9	1.7 (1.0–2.8)	1.3 (0.7–2.6)	0.8 (0.4–1.5)
Any sport injury requiring hospitalisation	73	46	25	**1.6 (1.4**–**1.9)**	**2.9 (1.9**–**4.4)**	**1.8 (1.2**–**2.7)**
Any surgery for sport injury	72	46	32	**1.6 (1.3**–**1.9)**	**2.2 (1.6**–**3.2)**	1.4 (1.0–2.1)
Injury that stopped participation in sport permanently	28	28	11	1.0 (0.7–1.4)	**2.5 (1.2**–**5.1)**	**2.5 (1.3**–**5.0)**

We have bolded the relative percentages that are > 1.43 or < 0.70 (at least a moderate difference between groups) for which the confidence intervals did not include a relative percentage of 1.0

*CLs* confidence limits

Compared with the non-contact sport group, the two rugby groups reported substantially more concussions per player (4.2 ± 4.2 for elite, 3.1 ± 3.5 for community, 0.4 ± 0.8 for non-contact). Cohen's *d* [95% confidence limits] were 1.1 [0.8–1.4] for elite rugby versus non-contact sport, 0.9 [0.6–1.1] for community rugby versus non-contact sport and 0.3 [0.1–0.5] for elite rugby versus community rugby. Analyses of self-rated health by sport group and self-reported concussions are provided in supplementary file 1, see electronic supplementary material [ESM]).

Current mood, medical conditions and health ratings of former player groups are presented in Table 3. Thirty-seven percent of former elite rugby players reported that they had osteoarthritis, compared with 18% of former community rugby players and 5.6% of non-contact sports people. There were no other substantial/meaningful differences in current physical health between groups.

**Table 3:** Key current physical health and fitness, mental health and social health characteristics of former player groups

	Elite rugby		Community rugby		Non-contact sport		Elite rugby versus community rugby	Elite rugby versus non-contact sport	Community rugby versus non-contact sport
HEALTH numeric variables	*n*	Mean (SD)	*n*	Mean (SD)	*n*	Mean (SD)	Cohen's *d* (95% confidence limits)		
Overall health rating	125	3.9 (0.8)	254	3.8 (0.8)	72	4.1 (0.9)	0.1 (− 0.1 to 0.3)	− 0.2 (− 0.5 to 0.1)	− 0.4 (− 0.6 to − 0.2)
Physical health rating	123	3.9 (0.9)	248	3.8 (0.9)	70	4.1 (0.8)	0.1 (− 0.1 to 0.3)	− 0.2 (− 0.5 to 0.1)	− 0.3 (− 0.5 to − 0.0)
Physical fitness health rating	123	3.5 (0.9)	247	3.3 (0.9)	71	3.5 (0.9)	0.2 (0.0 to 0.4)	0.0 (-0.3 to 0.3)	− 0.2 (− 0.4 to 0.0)
Diet/nutritional health rating	123	3.6 (0.9)	245	3.4 (0.9)	71	3.7 (0.7)	0.3 (0.0 to 0.5)	− 0.1 (− 0.4 to 0.2)	− 0.3 (− 0.6 to − 0.1)
Mental/psychological health rating	123	3.9 (0.9)	248	3.8 (0.9)	69	4.0 (0.8)	0.1 (− 0.1 to 0.3)	− 0.1 (− 0.4 to 0.2)	− 0.2 (− 0.4 to 0.0)
Exercise intensity	124	6.1 (2.3)	264	5.3 (2.5)	72	6.2 (2.3)	0.3 (0.1 to 0.5)	− 0.0 (− 0.3 to 0.3)	− 0.3 (− 0.6 to − 0.1)

HEALTH percentage variables	*n*	%	*n*	%	*n*	%	Relative percentage (95% confidence limits)		
Cardiovascular disease	127	2.4	271	0.7	72	2.8	3.2 (0.5 to 18.9)	0.9 (0.2 to 5.0)	0.3 (0.0 to 1.9)
Anxiety	127	4.7	271	7.7	72	6.9	0.6 (0.3 to 1.5)	0.7 (0.2 to 2.2)	1.1 (0.4 to 2.9)
Osteoarthritis	127	37	271	18.1	72	5.6	**2.0 (1.4 to 2.8)**	**6.5 (2.4 to 17.4)**	**3.3 (1.2 to 8.7**)
Chronic fatigue	127	0	271	3.3	72	2.8			1.2 (0.3 to 5.4)
Depression	127	11	271	10	72	12.5	1.1 (0.6 to 2.0)	0.9 (0.4 to 1.9)	0.8 (0.4 to 1.6)
Diabetes	127	2.4	271	3.3	72	2.8	0.7 (0.2 to 2.6)	0.9 (0.2 to 5.0)	1.2 (0.3 to 5.4)
High blood pressure	127	14.2	271	9.2	72	5.6	1.5 (0.9 to 2.7)	2.6 (0.9 to 7.3)	1.7 (0.6 to 4.6)
High cholesterol	127	16.5	271	15.1	72	16.7	1.1 (0.7 to 1.8)	1.0 (0.5 to 1.9)	0.9 (0.5 to 1.6)
Regular headaches/migraines	127	11	271	12.9	72	11.1	0.9 (0.5 to 1.5)	1.0 (0.4 to 2.3)	1.2 (0.6 to 2.4)
AUDIT Score ≥ 8 (hazardous drinking)	123	38	253	40	72	25	1.0 (0.7 to 1.2)	1.5 (1.0 to 2.4)	**1.6 (1.1 to 2.5)**
Current tobacco use	124	6.5	252	9.9	72	9.7	0.7 (0.3 to 1.4)	0.7 (0.3 to 1.8)	1.0 (0.5 to 2.3)
Any use of cannabis (lifetime)	122	34	245	44	70	43	0.8 (0.6 to 1.0)	0.8 (0.6 to 1.2)	1.0 (0.8 to 1.4)
Any use of other illegal recreational drugs (lifetime)	127	29	271	26	72	29	1.1 (0.8 to 1.6)	1.0 (0.6 to 1.6)	0.9 (0.6 to 1.3)

MOOD numeric variables	*n*	Mean (SD)	*n*	Mean (SD)	*n*	Mean (SD)	Cohen's *d* (95% confidence limits)		
Negative affect	123	17.3 (10.8)	243	17.8 (9.6)	70	19.4 (9.3)	− 0.1 (− 0.3 to 0.2)	− 0.1 (− 0.4 to 0.2)	− 0.2 (− 0.5 to 0.1)
Spielberger anger in	122	14.3 (3.3)	246	14.8 (3.7)	70	14.0 (3.3)	− 0.2 (− 0.4 to 0.0)	− 0.1 (− 0.2 to 0.4)	0.2 (− 0.0 to 0.5)
Spielberger anger out	122	12.6 (2.8)	246	12.7 (2.9)	70	12.4 (3.3)	− 0.1 (− 0.3 to 0.1)	0.0 (− 0.2 to 0.3)	0.1 (− 0.1 to 0.3)

ALCOHOL USE numeric variables	*n*	Mean (SD)	*n*	Mean (SD)	*n*	Mean (SD)	Cohen's *d* (95% confidence limits)		
AUDIT Consumption	123	4.7 (2.5)	253	5.1 (2.3)	72	4.7 (2.4)	− 0.2 (− 0.4 to 0.0)	0.0 (− 0.3 to 0.3)	0.2 (− 0.0 to 0.4)
AUDIT Dependence	123	0.4 (0.7)	252	0.5 (0.9)	72	0.3 (0.9)	− 0.2 (− 0.4 to 0.0)	0.1 (− 0.2 to 0.4)	0.2 (− 0.1 to 0.4)
AUDIT Problems	123	1.5 (2.0)	253	1.7 (2.4)	72	1.1 (1.8)	− 0.1 (− 0.3 to 0.1)	0.2 (0.0 to 0.5)	0.3 (0.1 to 0.5)
AUDIT Sum	123	6.5 (4.3)	253	7.4 (4.5)	72	6.1 (4.1)	− 0.2 (− 0.4 to 0.0)	0.1 (− 0.2 to 0.4)	0.3 (0.1 to 0.5)

Data are reported as mean ± standard deviation or a percentage (%) of participants as indicated. We have bolded standardised differences in means that are > 0.6 (moderate effects or greater) and relative percentages that are > 1.43 or < 0.7 (at least a moderate difference between groups) for which the confidence intervals did not include a standardised mean difference of zero or a relative percentage of 1.0

The n is shown for the total group numbers who answered the question

*AUDIT* Alcohol Use Disorders Identification Test, *SD* standard deviation

There were no substantial differences in self-reported mood between the player groups. A greater percentage of the elite rugby (38.2%) and community rugby player (40.3%) groups reported that they currently drink alcohol at hazardous levels (AUDIT Score > 8) compared with the non-contact sport (25%) player group. Mean scores on the AUDIT subscales were similar across the playing groups.

## Discussion

The *NZ-RugbyHealth* general health e-questionnaire cross-sectional study provided a snapshot of the playing and injury history, current demographics and health characteristics of former rugby and non-contact sport players and deliberately used several of the same research instruments as employed in RIPP. Our exploratory study formed an important step in helping to identify health issues amongst former rugby and non-contact sport players.

### Impact Injuries and Potential for Osteoarthritis

The hypothesis that rugby players are at substantially higher risk of injury than non-contact sport participants was supported by the results of our study. The elite rugby player group was at systematically higher risk of sustaining injuries during their playing careers than the non-contact sports player group; higher percentages of the elite rugby group were hospitalised because of a sporting injury and had a surgery related to an injury that occurred during participation in their sport than either the community rugby group or the non-contact sport player group. The proportions of the types of self-reported injuries were consistent with medically diagnosed injuries due to playing rugby from 2005 to 2017 recorded via the NZ national Accident Compensation Corporation claims [20].

Injuries were also a greater factor in rugby players stopping playing their sport permanently; 28% of both the elite and community rugby groups stopped playing because of injury, compared with 11% of the non-contact sports players.

Compared with the general population, former athletes have been found to have relatively high rates of osteoarthritis in the joints of the lower limb [21] and hand [22]. In our study, the prevalence of osteoarthritis was 37% for former elite players, 18% for former community players and 6% for non-contact players. Using an identical question to establish the prevalence of osteoarthritis to that used in our study, the *UK-RugbyHealth* study [21] reported 51% for elite rugby players, 36% for amateur rugby players and 22% for non-contact sports people. A previous cross-sectional study of 259 former elite English male rugby players assessed osteoarthritis prevalence relative to 5186 participants in the English Longitudinal Study of Aging [4]. Sixty percent of former elite rugby players reported osteoarthritis. Despite the differences in reported prevalence of osteoarthritis by group across the studies, in each case former rugby players were found to be at higher risk of developing osteoarthritis. In our study, the relative percentage for elite players was 5.7 times that of non-contact-sports players, which was higher than in the *UK-RugbyHealth* study [21], where the prevalence of medically diagnosed osteoarthritis was 2.3 times higher among former elite rugby players than non-contact sports people, and higher than that found in the English Longitudinal Study of Aging [4], where the relative prevalence of osteoarthritis among the former rugby players was four times that of males drawn from the general population. While we do not know what to attribute these inter-study differences to, factors such as differences in typical game play and style, environmental conditions (pitch and weather), or the way the groups reported injuries may all have contributed.

### Head Impacts and Potential for Brain Health Issues

Since the data for the *NZ-RugbyHealth* study were collected, the effects of concussive and sub-concussive head impacts on brain health have received increased governmental [23], media [24, 25] and research attention [26–30]. Areas of focus have included neurodegenerative impairment [31–33] and potential chronic traumatic encephalography (CTE) [34–37], mood disorders [38–40], substance-use disorders [41] and disturbed motor control [42].

A systematic review of possible long-term effects of sports-related concussion in former athletes reported that multiple concussions appeared to be a risk factor for cognitive impairment and mental health problems in some individuals [43]. Despite a high number of concussions sustained by rugby players during their playing careers compared with the non-contact group, there were no notable differences between the player groups in current self-reported health status post-retirement in our study. Our high number of concussions for the rugby groups is consistent with results of a 2014/15 study [44] of 52 retired male Scottish international rugby players and 29 male controls.

In a 2018 review [45] of six papers [44, 46–50] that examined the influence of playing rugby on brain health later in life (including papers [47, 49] that reported data from the *NZ RugbyHealth* cohort), it was concluded that there was “modest objective evidence” of decreased neuropsychological function in former rugby players. For ‘brain health’ the review included measures of cognitive function across eight domains (attention, executive functioning, information processing, motor speed, verbal ability, verbal memory, visual memory and visuospatial ability). Therefore, it did not include information regarding specific diagnoses of neurological conditions such as Alzheimer’s or Parkinson’s disease (or severe mental health/psychiatric conditions). This is a gap in the literature that supports our recommendation for prospective longitudinal studies of brain health.

### Alcohol and Other Substance Use

Many participants in rugby drink large quantities of alcohol as part of the cultural rituals of the sport [51]. The misuse of alcohol is a risk factor for a variety of medical conditions and poor health outcomes in New Zealand [52, 53]. As well as being a Group 1 carcinogen [54], alcohol misuse is strongly associated with self-harm injuries and injuries to others [55]. Although heavy drinking has traditionally been associated with rugby [56], relatively few studies have attempted to systematically describe alcohol use in cohorts of rugby players. The use of the AUDIT to document drinking patterns among New Zealand rugby players first occurred in the RIPP study [11]. An AUDIT score of > 8 is indicative of hazardous alcohol use; the higher the score the more likely it becomes that the person is misusing alcohol (i.e. likely to be harmed or harming others by its use, or likely to be or become dependent) [11]. Although the prevalence of drinking behaviour at hazardous levels in our two former rugby groups (38% to 40%) was less than amongst the RIPP cohort (84%), former rugby players reported higher rates of hazardous drinking than former non-contact sport players (25%) and New Zealand males of the same age range in the wider population (26%) [57]. Hazardous drinking behaviour among retired rugby players has also been described internationally. A study describing alcohol use among players from France, Ireland and South Africa [5] reported prevalence rates of 62% for adverse alcohol behaviour.

Alcohol companies have a long history of sponsoring rugby union teams and competitions in New Zealand [58]. Alcohol marketing has been consistently linked to adolescents initiating drinking and engaging in binge drinking [59, 60]. In 2014, the New Zealand Ministerial Forum on Alcohol Advertising and Sponsorship recommended changes to sports sponsorship and advertising [61] and a 2017 editorial in the *New Zealand Medical Journal* [62] called for the New Zealand Government to ban alcohol advertising and sponsorship of sport. A quarter of a century after the authors of the RIPP study [9] called for research into the effects of alcohol advertising and sponsorship on the promotion of the observed drinking patterns, alcohol sponsorship remains a feature of the New Zealand rugby landscape and hazardous drinking practices are commonplace among a significant minority of former players. Alcohol sponsorship, and particularly the provision of free or discounted alcohol to teams, has been directly associated with hazardous drinking among sports people [63]. Multiple alcohol-related incidents involving New Zealand’s professional players resulted in New Zealand Rugby undertaking a ‘Respect and Responsibility Review’ in 2016, the report on which was published in September 2017 [64]. A number of recommendations regarding alcohol were made in the report, primarily around educating rugby participants about the effects and potential harms of alcohol use; removing sponsorship was not put forward as a solution to dealing with alcohol-related harm by New Zealand Rugby [65]. We acknowledge that many of the participants in the study would have been playing rugby prior to these recommendations about alcohol being made and were likely exposed to the culture at the time, and their drinking habits may have persisted.

Tobacco companies have not been permitted to sponsor sports events in New Zealand since 1995. In the case of tobacco, government intervention to ban advertising at, and sponsorship of, sports events followed recognition of the deleterious effects of smoking tobacco products on the health of users. Use of tobacco among the retired players in our study (both rugby groups and non-contact athletes) was lower than for New Zealand males of a similar age (23%) [66]. Considering the higher prevalence of hazardous drinking behaviour shown in former sports participants than the general population, and the increasing knowledge of the harmful health effects of alcohol [54, 55, 67], public health specialists and New Zealand media have called for a debate on whether it is ethically defensible for sports organisations to continue to accept alcohol sponsorship [63, 65, 68].

Substance abuse can increase among people with traumatic brain injury [41]. Given the rugby cohorts experienced more concussions than the non-contact group, greater recreational drug use might have been expected in the rugby than in the non-contact players. Except for alcohol, the percentages of participants in the two rugby groups who used any recreational drugs in their lifetime were like those reported in the non-contact group and for males of the same age range in the general New Zealand population [66, 69]. This is similar to the results of a review [70] that reported there were no significant changes in substance use behaviours after mild traumatic brain injury.

A significant association between adverse alcohol use and osteoarthritis (OR 1.8, 95% CI 1.2–2.6) has been reported in a group of former UK elite athletes (rugby, football, ice hockey, Gaelic sports and cricket) [71] and beer consumption has been associated with increased risk of knee or hip osteoarthritis in a case control study [72]. In rugby, it is likely that the sport predisposes athletes to osteoarthritis because of the joint injuries that are commonplace in the sport. Concurrently, they are drinking large amounts of alcohol [73]. While a cause and effect relationship between alcohol and osteoarthritis cannot be ruled out, it seems more probable that hazardous alcohol use and the development of osteoarthritis are independently associated with participation in rugby union.

### Limitations of the Study

#### Self-report Questionnaire and Item Composition

Data on health history including concussion were obtained from self-report, thus recall bias is a potential issue. The extent to which recall may be impaired, and whether any impairment differs systematically between groups, are important considerations when health histories are collected many years after exposure [74]. Factors such as frequency, duration and perceived significance of events appear to impact on the ability of survey respondents to recall them. The elite rugby or non-contact players, for whom physical performance was critical to success, may be more medically aware and knowledgeable than the community-level players or the general population, and any injuries they experienced may have had greater salience for them, thus they may have recalled and reported more injuries and illnesses. In addition, there is evidence that people tend to distort self-reported events in socially desirable directions, for example to under-report substance use [74, 75]. We did not examine whether, or to what extent, this was the case in our study.

We sought to reduce potential inter-responder variability by asking whether conditions had been medically diagnosed; even so, self-reported health issues do not necessarily align with medically diagnosed health issues [76]. Self-reporting of prior concussion is a reliable ordinal measure and may reflect some head injuries that were not previously reported [38, 77]. We did not assess the time since the last concussion, nor concussions that were not related to sport. Further research to examine medically diagnosed concussion history and associations with substance abuse, excessive alcohol use and current health is needed.

#### Methodology and Statistical Implications

Like all studies involving recruitment of volunteers, the study was subject to non-response bias. We originally planned to have 200 players in each group, but despite leaving the survey open for longer than originally intended and requesting (and obtaining) assistance for recruitment from NZ Players’ Associations, it was difficult at the time to recruit participants. The number of respondents in the elite rugby and non-contact groups were lower than intended, whereas we obtained more respondents than planned for the community rugby group.

The non-contact sport group that was added to enable sports groups with and without collisions to be compared, had 26% who had sustained a concussion. As this group consisted of a mix of elite (43%) and community level non-contact sport players, there could have been regression to the mean within that group that may have affected the magnitude of the differences observed between groups.

Non-response bias could have impacted the degree to which the samples are accurate representations of the wider population of players from which they were drawn. We were therefore conservative in interpreting the effects as representing important differences between the groups. Our interpretation of the standardised differences in means and the relative percentages highlighted in the tables is that there was a big enough difference between the groups to warrant follow-up with more robust research methods.

Large inter-individual variation was common in the variables, which means we may be missing important real effects that hold in the wider population. Conversely, in some instances, we may be identifying effects that look important but are statistical artefacts produced through chance or the biases to which the study was prone. Due to low numbers we were not able to determine any differences in Alzheimer’s disease or dementia.

Due to the cross-sectional nature of this study, causality between rugby/non-contact sport participation and demographics or general health in later life was unable to be determined. For example, we cannot eliminate the possibility that the former player groups may have differed in general health or demographic characteristics prior to participation in sport or from other aspects of their lives over their playing career. A strength of our study was that it enabled us to identify potential health issues associated with participation in rugby in a relatively short time span, to assist with planning of future follow-up studies. It remains unclear whether potential negative outcomes from injuries and alcohol use in rugby outweigh long-term health benefits associated with participation in physical activities per se. An interdisciplinary approach to the clinical care and support of former elite athletes after their careers has been advocated, as the interaction between physical and mental health issues occurring in the long term is complex [71].

For athletes to be able to make informed decisions about the implications of playing rugby on their long-term health, they need quality health outcome and risk factor data. Longitudinal research on the health of rugby athletes over extended follow-up periods is needed to examine changes in health over time while accounting for demographic and health risk covariates, and to address non-response bias. The relative youth of the groups (43.8 years on average) means that health issues that typically do not emerge until later life may not have yet manifested.

## Conclusions

Former rugby player groups were at higher risk than the non-contact player group for most injuries during their playing careers. Following retirement, substantially higher percentages of the rugby player groups reported having been diagnosed with osteoarthritis and engaging in hazardous alcohol consumption than the non-contact player group. In terms of medical conditions, mood and life satisfaction, there were no substantial differences between former rugby players and non-contact sports people. All former player groups had lower rates of tobacco use than males of similar age in New Zealand.

## Recommendations

Given the findings of the study, we recommend research to address implications for player general health in retirement from rugby, including:

- determining what the contributing factors to hazardous alcohol consumption are among rugby players/culture/environment;
- determining how players should be advised of the injuries they are likely to experience and the potential for longer term health issues related to these injuries, such as osteoarthritis, so they can make informed choices about engagement in rugby.

## Supplementary Information

Below is the link to the electronic supplementary material.

Supplementary file1 (PDF 426 KB)

## Declarations

### Data availability

As the study was funded by World Rugby and New Zealand Rugby, and the dataset is under embargo, the dataset and software analyses code for the research are not available unless a request is made to World Rugby.

### Provenance and peer review

Not commissioned, externally peer reviewed.

## References

[1] Yeoman C, Kenny IC, Cahalan R (2018). The incidence of injury in amateur male rugby union: a systematic review and meta-analysis. Sports Med.

[2] Kilic Ö, Hopley P, Kerkhoffs GMMJ (2019). Impact of concussion and severe musculoskeletal injuries on the onset of mental health symptoms in male professional rugby players: a 12-month study. BMJ Open Sport Exerc Med.

[3] Du Preez EJ, Graham KS, Gan TY (2017). Depression, anxiety, and alcohol use in elite rugby league players over a competitive season. Clin J Sport Med.

[4] Davies MAM, Judge AD, Delmestri A (2017). Health amongst former rugby union players: a cross-sectional study of morbidity and health related quality of life. Sci Rep.

[5] Gouttebarge V, Kerkhoffs G, Lambert M (2016). Prevalence and determinants of symptoms of common mental disorders in retired professional Rugby Union players. Eur J Sport Sci.

[6] Hume PA, Lewis G, Theadom A, et al. Player Fact Sheet: World Rugby/NZ Rugby/Auckland University of Technology RugbyHealth project. 12th May 2015 2015. Auckland: Sport Performance Research Institute New Zealand, Auckland University of Technology, New Zealand.

[7] Quarrie KL, Alsop JC, Waller AE, et al. The New Zealand rugby injury and performance project. VI. A prospective cohort study of risk factors for injury in rugby union football. Department of Orthopaedics and Sports Medicine, Seattle Children's Hospital, 4800 Sand Point Way NE, Seattle, WA 98105, USA. 2001; 35: 157–166. 2001/05/29. 10.1136/bjsm.35.3.157. 10.1136/bjsm.35.3.157 PMC1724329 11375873

[8] Bird YN, Waller AE, Marshall SW, et al. The New Zealand Rugby Injury and Performance Project: V. Epidemiology of a season of rugby injury. Department of Orthopaedics and Sports Medicine, Seattle Children's Hospital, 4800 Sand Point Way NE, Seattle, WA 98105, USA. 1998; 32: 319–325. 1998/12/29. 10.1136/bjsm.32.4.319. 10.1136/bjsm.32.4.319 PMC1756118 9865405

[9] Waller AE, Feehan M, Marshall SW, et al. The New Zealand Rugby Injury and Performance Project: I. Design and methodology of a prospective follow-up study. Department of Orthopaedics and Sports Medicine, Seattle Children's Hospital, 4800 Sand Point Way NE, Seattle, WA 98105, USA. 1994; 28: 223–228. 1994/12/01. DOI: 10.1136/bjsm.28.4.223. 10.1136/bjsm.28.4.223 PMC1332080 7894951

[10] Gerrard DF, Waller AE and Bird YN. The New Zealand Rugby Injury and Performance Project: II. Previous injury experience of a rugby-playing cohort. Department of Orthopaedics and Sports Medicine, Seattle Children's Hospital, 4800 Sand Point Way NE, Seattle, WA 98105, USA. 1994; 28: 229–233. 1994/12/01. 10.1136/bjsm.28.4.229. 10.1136/bjsm.28.4.229 PMC1332081 7894952

[11] Quarrie KL, Feehan M, Waller AE (1996). The New Zealand Rugby injury and performance project: alcohol use patterns within a cohort of rugby players. Addiction.

[12] Hume P, Potts G (1999). SportSmart: the 10-point plan for sports injury prevention: an educational resource.

[13] Quarrie KM, Gianotti S, Hopkins WG (2007). Effect of nationwide injury prevention programme on serious spinal injuries in New Zealand rugby union. Brit Med J.

[14] Simpson J, Chalmers D, Waller A (1994). Tackling rugby injury: recommendations for reducing injury to rugby union players in New Zealand.

[15] Tennant R, Joseph S, Stewart-Brown S (2007). The affectometer 2: a measure of positive mental health in UK populations. Qual Life Res.

[16] Knight RG, Chisholm BJ, Paulin JM (1988). The Spielberger Anger Expression Scale: some psychometric data. Br J Clin Psychol.

[17] Saunders JB, Aasland OG, Babor TF (1993). Development of the Alcohol Use Disorders Identification Test (AUDIT): WHO collaborative project on early detection of persons with harmful alcohol consumption–II. Addiction.

[18] Dawson DA, Grant BF, Stinson FS (2005). Effectiveness of the derived alcohol use disorders identification test (AUDIT-C) in screening for alcohol use disorders and risk drinking in the general population. Alcoholism Clin Exper Res.

[19] Hopkins WG. A scale of magnitudes for effect statistics; 2002. http://www.sportsci.org/resource/stats/effectmag.html. Accessed 26 Jan 2009.

[20] Quarrie K, Gianotti S, Murphy I (2019). Injury risk in New Zealand rugby union: a nationwide study of injury insurance claims from 2005 to 2017. Sports Med.

[21] Hind K, Konerth N, Entwistle I (2020). Cumulative sport-related injuries and longer term impact in retired male elite- and amateur-level rugby code athletes and non-contact athletes: a retrospective study. Sports Med.

[22] Mary EJ, Madeleine AMD, Karishma S, Simon K, Nick Peircec KML, Keith AS, Andrew DJ, Julia LN, Nigel KA (2019). The prevalence of hand and wrist osteoarthritis in elite former cricket and rugby union players. J Sci Med Sport.

[23] UK parliament. Concussion in sport. In: House of Commons Digital C, Media and Sport Committee, (ed.). England: House of Commons, 2021, p. 42.

[24] RugbyPass. Concussion claims another—RugbyPass looks at players who've been forced to retire due to head knocks. 2018. https://www.rugbypass.com/news/concussion-claims-another-rugbypass-looks-players-whove-forced-retire-due-head-knocks/. Accessed 14 Feb 2018.

[25] RW Staff. World Rugby facing concussion lawsuit. 2020. https://www.rugbyworld.com/news/world-rugby-facing-concussion-lawsuit-118095. Accessed 8 Dec 2020.

[26] Alberts JL, Linder SM (2015). The utilization of biomechanics to understand and manage the acute and long-term effects of concussion. Kinesiol Rev.

[27] DeKosky S, Ikonomovic M, Gandy S (2010). Traumatic brain injury—football, warfare, and long-term effects. N Engl J Med.

[28] King DA, Hume PA, Clark TN (2021). Use of RESTQ-Sport and King–Devick test to monitor changes during recovery of concussion in an amateur women’s rugby union team. JSM Phys Med Rehabilit.

[29] King D, Hume PA, Gissane C (2017). Semi-professional rugby league players have higher concussion risk than professional or amateur participants: a pooled analysis. Sports Med.

[30] King D, Hume PA, Gissane C (2016). The influence of head impact threshold for reporting data in contact and collision sports: systematic review and original data analysis. Sports Med.

[31] De Beaumont L, Theoret H, Mongeon D, et al. Brain function decline in healthy retired athletes who sustained their last sports concussion in early adulthood. Brain. 2009; 132: 695–708. Research Support, Non-U.S. Gov't 2009/01/30. 10.1093/brain/awn347. 10.1093/brain/awn347 19176544

[32] Guskiewicz KM, Marshall SW, Bailes J (2005). Association between recurrent concussion and late-life cognitive impairment in retired professional football players. Neurosurgery.

[33] Pearce AJ, Hoy K, Rogers MA, Corp DT, Davies CB, Maller JJ, Fitzgerald PB (2015). Acute motor, neurocognitive and neurophysiological change following concussion injury in Australian amateur football. A prospective multimodal investigation. J Sci Med Sport.

[34] Cantu RC (2007). Chronic traumatic encephalopathy in the National Football League. Neurosurgery.

[35] McKee AC, Cantu RC, Nowinski CJ (2009). Chronic traumatic encephalopathy inathletes: progressive tauopathy after repetitive head injury. J Neuropathol ExpNeuro.

[36] McCrory P, Meeuwisse WH, Kutcher JS (2013). What is the evidence for chronic concussion-related changes in retired athletes: behavioural, pathological and clinical outcomes?. Br J Sports Med.

[37] Carson AJ (2017). Concussion, dementia and CTE: are we getting it very wrong?. J Neurol Neurosurg Psychiatry.

[38] Kerr Z, Marshall S, Harding HJ (2012). Nine-year risk of depression diagnosis increases with increasing self-reported concussions in retired professional football players. Am J Sport Med.

[39] Schwenk TL, Gorenflo DW, Dopp RR, et al. Depression and pain in retired professional football players. *Med Sci Sports Exerc* 2007; 39: 599–605. Research Support, Non-U.S. Gov't 2007/04/07. 10.1249/mss.0b013e31802fa679. 10.1249/mss.0b013e31802fa679 17414796

[40] Guskiewicz KM, Marshall SW, Bailes J (2007). Recurrent concussion and risk of depression in retired professional football players. Med Sci Sport Exer.

[41] West S (2011). Substance abuse among persons with traumatic brain injury: a review. Neurorehabilit Neural Repair.

[42] De Beaumont L, Tremblay S, Henry LC, et al. Motor system alterations in retired former athletes: the role of aging and concussion history. BMC Neurol. 2013; 13: 109. http://www.biomedcentral.com/1471-2377/13/109. 10.1186/1471-2377-13-109 PMC3765614 23972282

[43] Manley GT, Gardner AJ, Schneider KJ (2017). A systematic review of potential long-term effects of sport-related concussion. Br J Sports Med.

[44] McMillan TM, McSkimming P, Wainman-Lefley J (2017). Long-term health outcomes after exposure to repeated concussion in elite level rugby union players. J Neurol Neurosurg Psychiatry.

[45] Cunningham J, Broglio S, Wilson F (2018). Influence of playing rugby on longterm brain health following retirement: a systematic review and narrative synthesis. BMJ Open Sport Exerc Med.

[46] Thornton AE, Cox DN, Whitfield K (2008). Cumulative concussion exposure in rugby players: neurocognitive and symptomatic outcomes. J Clin Exp Neuropsychol.

[47] Hume PA, Theadom A, Lewis G (2016). A comparison of cognitive function in former rugby union players compared to former non-contact sport players and the impact of concussion history. Sports Med.

[48] Decq P, Gault N, Blandeau M (2016). Long-term consequences of recurrent sports concussion. Acta Neurochir.

[49] Lewis GN, Hume PA, Stravric V (2017). NZ Rugby Health study: Motor cortex excitability in retired elite and community level rugby players. N Z Med J.

[50] Gardner AJ, Iverson GL, Wojtowicz M (2017). MR spectroscopy findings in retired professional rugby league players. Int J Sports Med.

[51] Kahu-Kauika PS (2011). Exploring players' perceptions about alcohol: the impact of alcohol on the rugby team culture.

[52] Ministry of Health (2012). The Health of New Zealand Adults 2011/12: key findings of the New Zealand Health Survey.

[53] Szabo A, Towers A, Sheridan J (2021). Ten-year trajectories of alcohol consumption in older adult New Zealanders. J Gerontol B Psychol Sci Soc Sci.

[54] Rumgay H, Shield K, Charvat H (2021). Global burden of cancer in 2020 attributable to alcohol consumption: a population-based study. Lancet Oncol.

[55] Rehm J, Mathers C, Popova S (2009). Alcohol and Global Health 1: Global burden of disease and injury and economic cost attributable to alcohol use and alcohol-use disorders. Lancet.

[56] Phillips J (1987). A Man's country? The Image of the Pakeha Male, a History.

[57] Ministry of Health. Hazardous drinking in 2011/12: findings from the New Zealand Health Survey. 2013.

[58] Gee S, Batty R (2020). Alcohol sponsorship and New Zealand Regional Rugby Unions: crisis point or business as usual?. Int J Sociol Leisure.

[59] Anderson P, De Bruijn A, Angus K (2009). Impact of alcohol advertising and media exposure on adolescent alcohol use: a systematic review of longitudinal studies. Alcohol Alcohol.

[60] Jernigan D, Noel J, Landon J (2017). Alcohol marketing and youth alcohol consumption: a systematic review of longitudinal studies published since 2008. Addiction.

[61] Ministerial Forum on Alcohol Advertising and Sponsorship (2014). Ministerial forum on alcohol advertising and sponsorship: recommendations on alcohol advertising and sponsorship.

[62] O’Brien KS, Chikritzhs T (2017). Time for the New Zealand government to ban alcohol advertising and sponsorship in sport. N Z Med J.

[63] O’Brien KS, Kypri K (2008). Alcohol industry sponsorship and hazardous drinking among sportspeople. Addiction.

[64] New Zealand Rugby. Respect and responsibility review. 2017.

[65] Radio New Zealand. Alcohol and rugby: 'Education doesn't change behaviour'. 2017.

[66] Ministry of Health (2014). Tobacco Use 2012/13: New Zealand Health Survey.

[67] Brown K (2016). Association between alcohol sports sponsorship and consumption: A systematic review. Alcohol Alcohol.

[68] Opinion: The link between alcohol and rugby. Whanganui Chronicle; 2021.

[69] Ministry of Health. Drug Use in New Zealand: Key Results of the 2007/08 New Zealand Alcohol and Drug Use Survey. Ministry of Health; 2010.

[70] VanderVeen JD (2021). TBI as a risk factor for substance use behaviors: a meta-analysis. Arch Phys Med Rehabil.

[71] Schuring N, Aoki H, Gray J (2017). Osteoarthritis is associated with symptoms of common mental disorders among former elite athletes. Knee Surg Sports Traumatol Arthrosc.

[72] Muthuri SG, Zhang W, Maciewicz RA (2015). Beer and wine consumption and risk of knee or hip osteoarthritis: a case control study. Arthritis Res Ther.

[73] Zhou J, O’Brien KS, Heim D (2013). Alcohol consumption in sportspeople: the role of social cohesion, identity and happiness. Int Rev Sociol Sport.

[74] Coughlin SS (1990). Recall bias in epidemiologic studies. J Clin Epidemiol.

[75] Davis CG, Thake J, Vilhena-Churchill N (2010). Social desirability biases in selfreported alcohol consumption and harms. Addict Behav.

[76] Kerr ZY, Mihalik JP, Guskiewicz KM (2015). Agreement between athlete-recalled and clinically documented concussion histories in former collegiate athletes. Am J Sports Med.

[77] Kerr Z, Marshall S, Guskiewicz K (2012). Reliability of concussion history in former professional football players. Med Sci Sport Exerc.

