# Supplementary material for: NZ-RugbyHealth Study: Self-reported Injury Experience and Current Health of Former Rugby Union and Non-contact Sport Players
Source: Sports Med. 2022 Jan 28;52(7):1701–13. doi: 10.1007/s40279-021-01630-7 (PMC9213390; doi:10.1007/s40279-021-01630-7)
Supplement: Supplementary file 1 — Supplementary file1 (PDF 426 KB) [file 40279_2021_1630_MOESM1_ESM.pdf]

NZ-RUGBYHEALTH STUDY: SELF-REPORTED INJURY EXPERIENCE AND CURRENT HEALTH OF FORMER RUGBY UNION AND NON-CONTACT SPORT PLAYERS

Short title/running head: Injury history and health of former rugby and non-contact sport players.

Patria A Hume[*1,2], Kenneth L Quarrie[4,1], Gwyn N Lewis[3], Alice Theadom[2]

[1] Sports Performance Research Institute New Zealand (SPRINZ), Auckland University of Technology, Auckland, New Zealand
[2] Traumatic Brain Injury Network (TBIN), Auckland University of Technology, Auckland, New Zealand
[3] Health and Rehabilitation Research Institute (HRRI), Auckland University of Technology, Auckland, New Zealand
[4] New Zealand Rugby, Wellington, New Zealand

*Author for correspondence:
Professor Patria Hume PhD
Sport Performance Research Institute New Zealand, Faculty of Health and Environmental Sciences, Auckland University of Technology, Private Bag 92006, Auckland 1142, New Zealand
Email patria.hume@aut.ac.nz

Electronic Supplementary Materials: 1 (Health ratings by group and concussion experience)

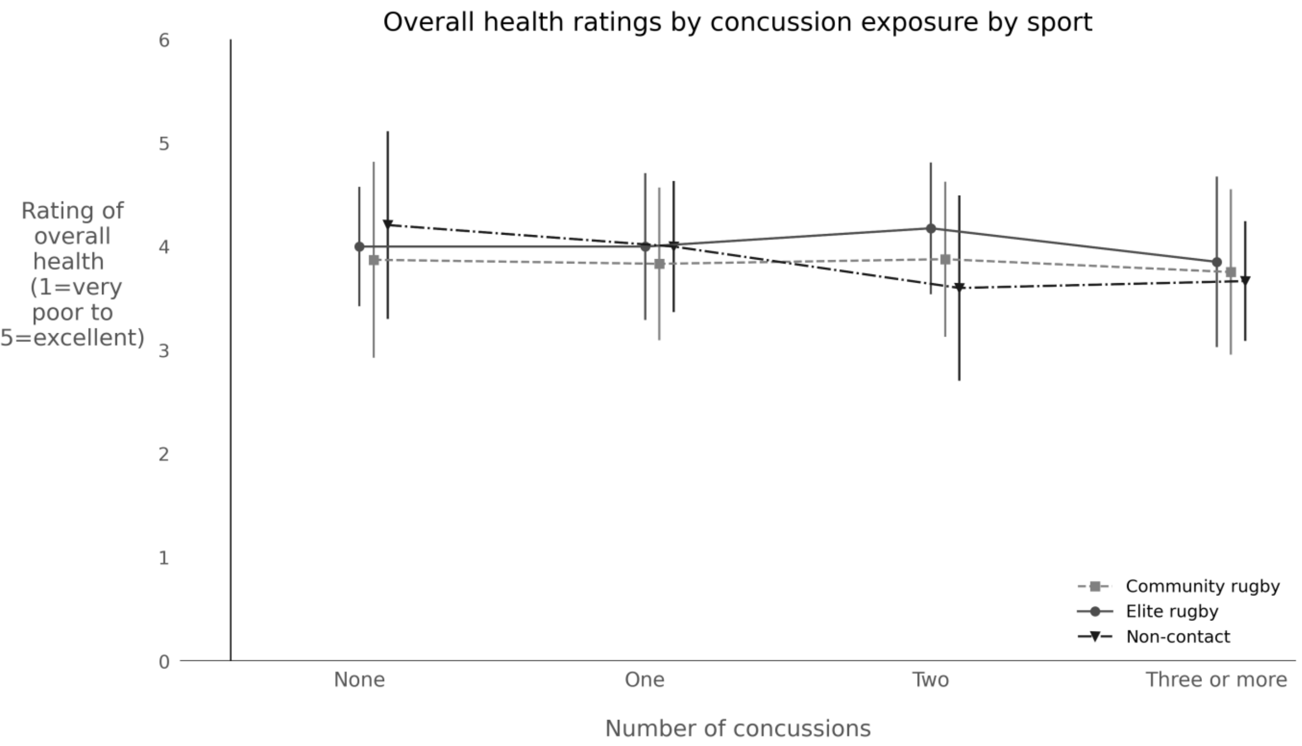
Overall health ratings by concussion exposure by sport
Rating of overall health (1=very poor to 5=excellent)
0
1
2
3
4
5
6
None
One
Two
Three or more
Number of concussions
Community rugby
Elite rugby
Non-contact

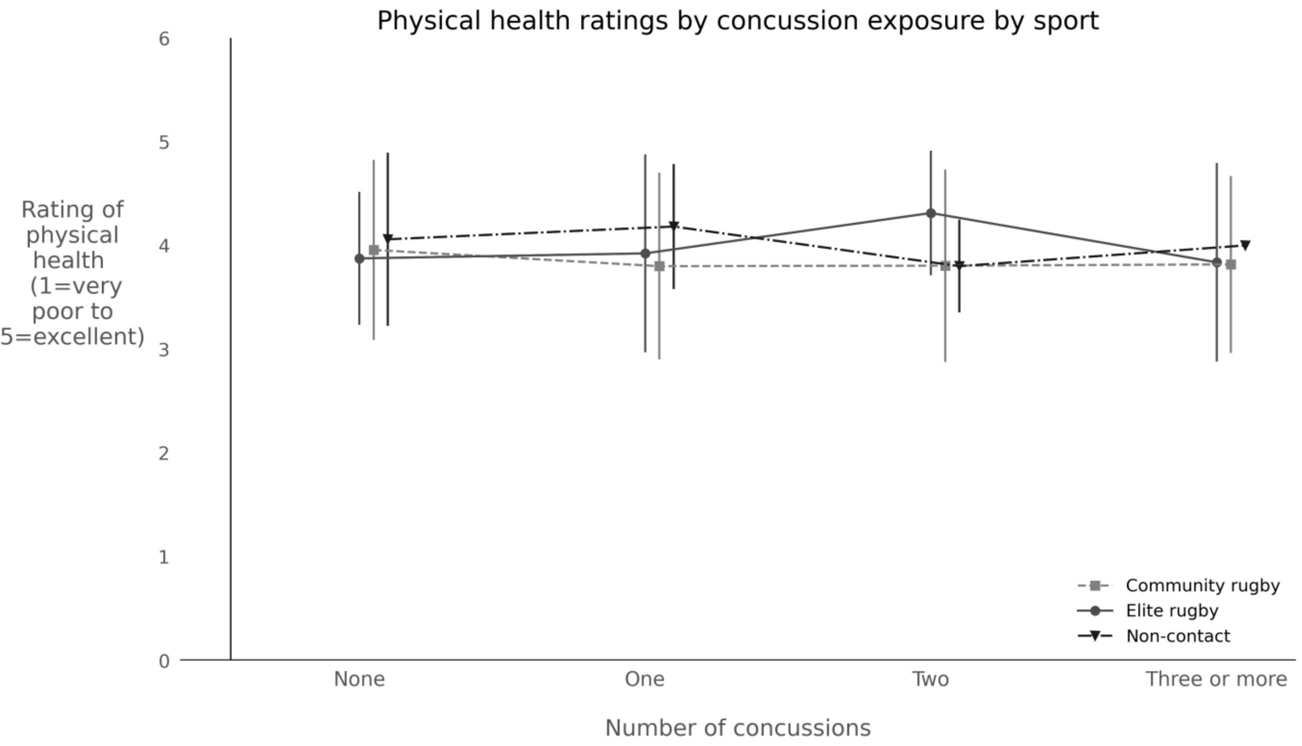
Physical health ratings by concussion exposure by sport
Rating of physical health (1=very poor to 5=excellent)
0
1
2
3
4
5
6
None
One
Two
Three or more
Number of concussions
Community rugby
Elite rugby
Non-contact

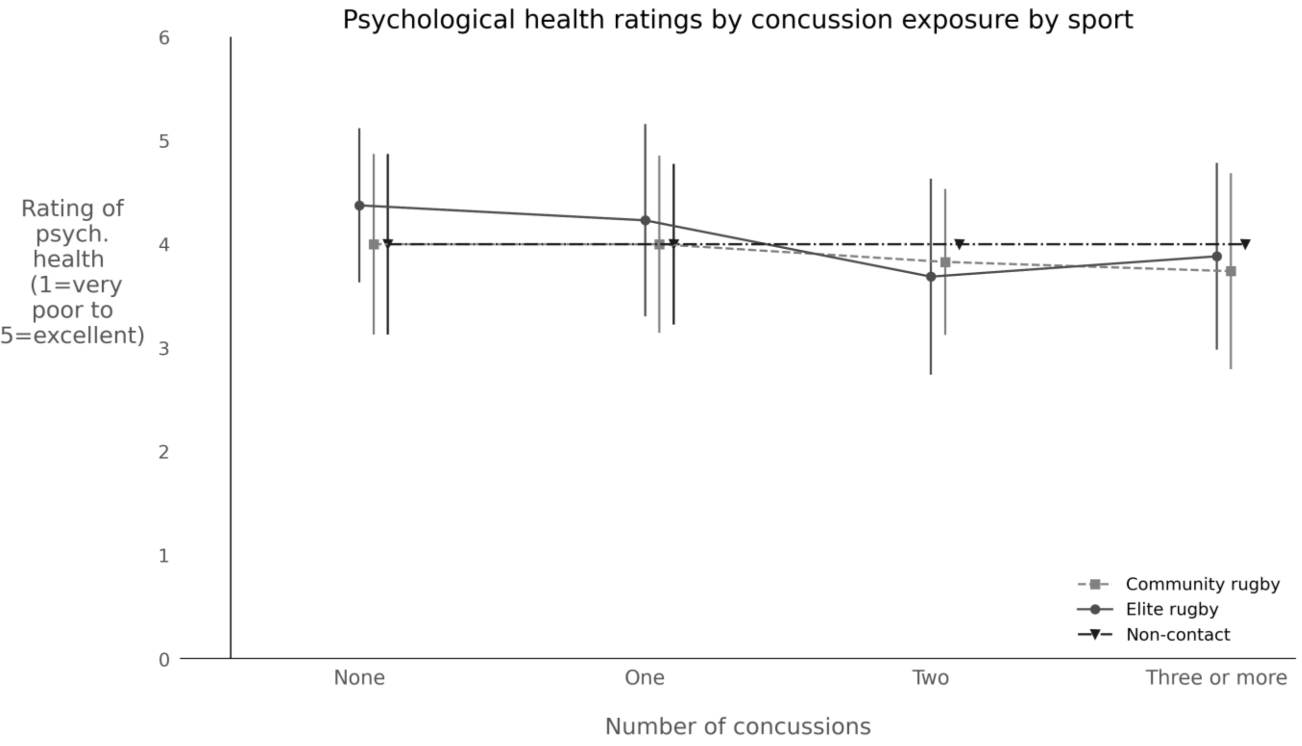
Psychological health ratings by concussion exposure by sport
Rating of psych. health (1=very poor to 5=excellent)
0
1
2
3
4
5
6
None
One
Two
Three or more
Number of concussions
Community rugby
Elite rugby
Non-contact

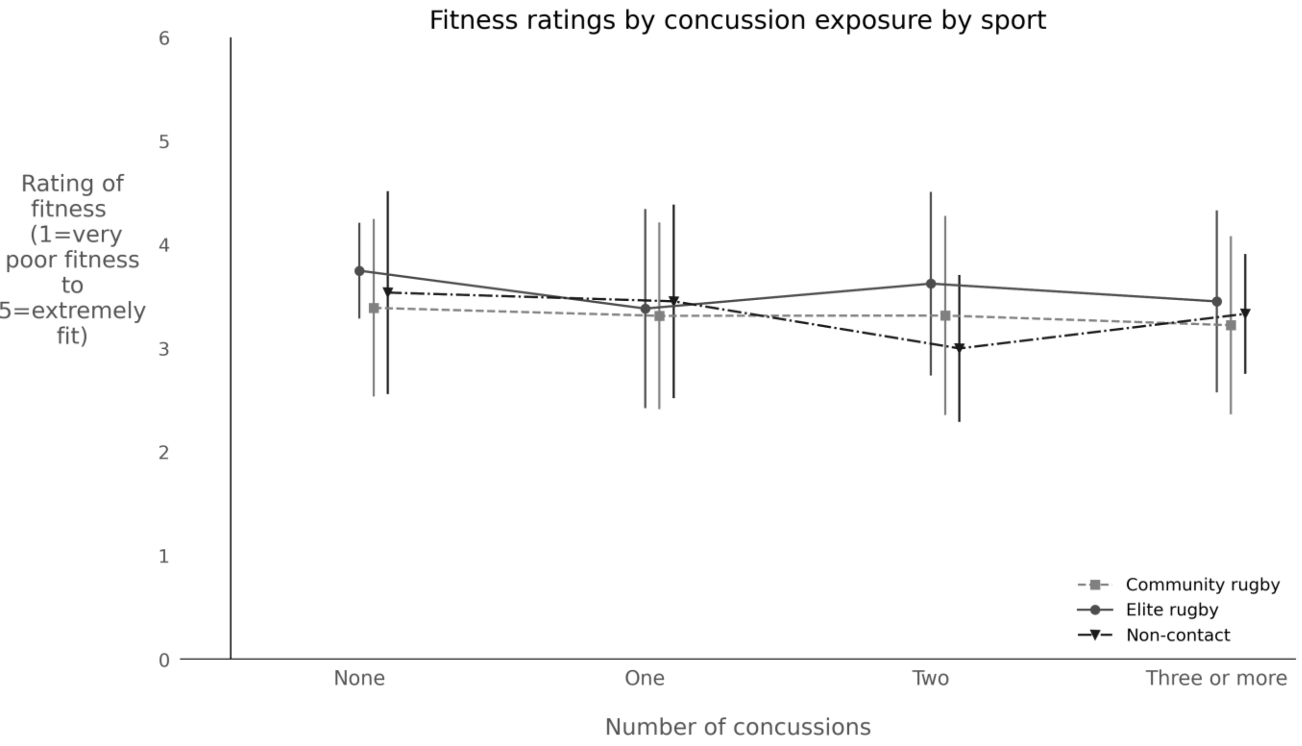

Fitness ratings by concussion exposure by sport
Rating of fitness (1=very poor fitness to 5=extremely fit)
0
1
2
3
4
5
6
None
One
Two
Three or more
Number of concussions
Community rugby
Elite rugby
Non-contact

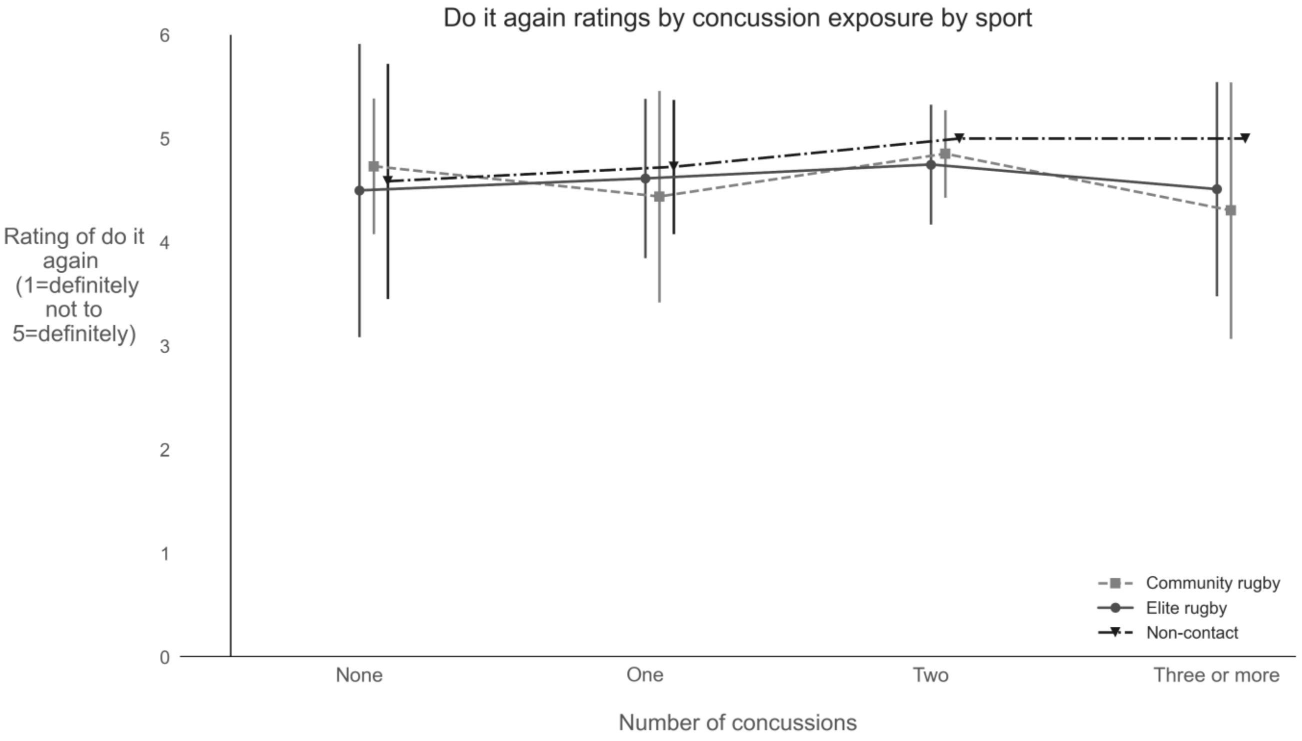

Do it again ratings by concussion exposure by sport
Rating of do it again (1=definitely not to 5=definitely)
0
1
2
3
4
5
6
None
One
Two
Three or more
Number of concussions
Community rugby
Elite rugby
Non-contact
